# Tolerance of an Aquatic Power Training Program by Older Adults with Symptomatic Knee Osteoarthritis

**DOI:** 10.1155/2012/895495

**Published:** 2012-09-13

**Authors:** Neil A. Segal, Robert Wallace

**Affiliations:** ^1^Departments of Orthopaedics & Rehabilitation, Radiology and Epidemiology, The University of Iowa, 200 Hawkins Drive, 0728 JPP, Iowa City, IA 52242-1088, USA; ^2^Department of Epidemiology, The University of Iowa, 200 Hawkins Drive, 0728 JPP, Iowa City, IA 52242-1088, USA

## Abstract

*Objective*. To determine the tolerance and feasibility of aquatic-based power training for improving lower limb muscle power, impairments, and mobility in adults with symptomatic knee OA. *Participants*. Twenty-nine adults, age 50 years and over, with symptomatic knee OA (ACR clinical criteria) and mobility limitation (400-meter walk time slower than median for sex and decade) completed 45-minute aquatic power training sessions twice weekly for 6 weeks. *Main Outcome Measurements*. Prospective outcomes included tolerance of the program, as well as change in stair climb power, 400-meter walk time, overall and knee-specific pain, activities of daily living (ADL), quality of life (QOL), and lower limb function at 6- and 12-week follow-up. *Results*. The training intensity required modification for 9 of the 29 participants. Lower limb muscle power, ADL, QOL, and overall pain were improved immediately and 6 weeks following completion (all *P* < 0.05). However, 400-meter walk times, and lower limb function did not differ from baseline. *Conclusions*. A 6-week aquatic rehabilitation program appears to be well tolerated by adults with symptomatic knee OA with mobility limitations and may result in improved lower limb muscle power, symptoms, ADL, and QOL. However, this intervention may have insufficient specificity or intensity for improving physical function.

## 1. Introduction

The 25 million Americans suffering from osteoarthritis (OA) represent a significant social and economic burden [1], and this number is projected to reach 67 million by 2030 [2]. The knee is the weight-bearing joint most commonly affected [3], leading to significant mobility limitations. In fact, OA accounts for more functional limitations in walking and stair climbing than any other musculoskeletal disease [4], and these limitations are associated with physical dependence and an earlier death.

In the context of knee OA, pain and low muscle power (the product of force and velocity) lead to functional limitations such as reduced community mobility. Reduced activity due to these factors may lead to further weakness and altered cartilage nutrition, contributing to further joint pathology, impairments, functional limitations, and disability. In particular, velocity of muscle contraction declines with aging, leading to a more precipitous drop in muscular power than in strength [5, 6]. The higher rate of loss of power than strength in the knee extensors [7] and plantar flexors [8] may have important functional implications, considering the importance for standing, walking, and ascending stairs [9]. It is therefore not surprising that muscle power correlates better with functional limitations in older adults than does strength [7, 10–12]. This suggests that power training may be useful for preserving mobility in older adults.

In older adults without symptomatic knee OA, a land-based weighted vest program has been shown to be well tolerated and effective in improving lower limb muscle power and chair rise speed [13], as well as physical performance measures predictive of disability, institutionalization, and mortality [14]. Prior work in our lab suggested that a similar program improves stair climb time in older adults with symptomatic knee OA, but approximately half of the participants were unable to tolerate the intervention—either could not use any weight in the vest or discontinued participation [15]. It also is important to consider that knee OA may lead to symptoms that prevent activation of muscles at high velocities during weight bearing. Thus, although power training has the potential to improve mobility, translating these findings to the population of interest will require that the training be tolerable in a greater proportion of older adults with symptomatic knee OA. 

A solution to the problem of delivering power training to those with symptomatic knee OA may be to deliver it in an aquatic environment, as submersion in water is known to reduce knee symptoms [16–18] and improve knee range of motion, although prior studies did not employ power training [19]. The properties of water—buoyancy, viscosity (leading to turbulence and drag), specific heat, and hydrostatic pressure—allow aquatic training to be well tolerated [16, 18], due to the upward force of buoyancy reducing weight bearing and viscosity increasing resistance to exercise movements, and therefore may enable an exercise intensity sufficient to increase lower limb muscle power. For power training, the load and velocity of movement determine the intensity. The viscosity of the aquatic environment (resistance to adjacent fluid layers sliding freely) causes velocity-dependent resistance to body movements by means of turbulence and drag. Due to turbulence, resistance to movement is proportional to the velocity squared and eddy currents trail behind the moving body part, creating drag that further increases the resistance. There is also evidence to suggest that aquatic resistance training may improve neural activation of the quadriceps and hamstrings [20], offering additional benefit.

Consequently, patients with symptomatic knee OA may be able to tolerate power training in an aquatic environment better than in a land-based environment. However, there have been mixed results regarding the effectiveness of exercise in an aquatic environment. A 12-week aquatic strength-training program in 55–75-year-old individuals with unilateral knee arthroplasty was well tolerated and significantly improved knee extensor and flexor power, thigh muscle cross-sectional areas, and stair climb and walking times compared with those randomized to control [21], although only the effects on muscle power persisted at 12-month followup [22]. However, other randomized, controlled trials of individuals age 50 and older with lower limb osteoarthritis revealed that, in comparison with a control group, walking times and distance improved with either an aquatic or a gym-based intervention in a 6-week study [23], while in an 8-week study, land-based training, but not aquatic-based training significantly improved isokinetic muscle strength [24]. Other investigators have reported an improvement in pain but no changes in quadriceps strength with aquatic-based therapy for older adults with knee or hip OA [17, 18], while others have not detected differences in pain, comparing aquatic-based versus land-based training [16, 25]. 

Thus, although evidence suggests that *resistance* training may be well tolerated and improve physical function [17, 26, 27], and reduce fall risk [28], it is currently unknown whether *power* training may be a promising means of improving mobility and lower limb muscle power and mobility limitations in older adults with symptomatic knee OA [26]. Previous aquatic-based interventional studies did not use higher-velocity exercises to focus training on improving lower limb power. Given the lack of tolerable and effective power training programs for older adults with symptomatic knee OA and the tolerance of nonpower training in the aquatic environment, this study was initiated (1) to explore whether aquatic-based power training would be tolerable as well as (2) to explore whether a novel 6-week aquatic-based power training program would improve lower limb muscle power and reduce lower limb mobility limitations (400-meter walk time) and knee pain in adults over age 50 with symptomatic knee OA both immediately and 6 weeks following completion of the program.

## 2. Materials and Methods

### 2.1. Subject Recruitment

This study enrolled adults age 50 and older, residing within commuting distance of our institution, with frequent knee symptoms (pain, aching, or stiffness) on most of the last 30 days, knee OA by ACR clinical criteria (using history and physical examination to determine the presence of pain in the knee plus three of the following: over 50 years of age, less than 30 minutes of morning stiffness, crepitus on active motion, bony tenderness, bony enlargement, and no palpable warmth of synovium) [29], and mobility limitation. Mobility limitation was defined by a 400-meter walk time slower than the median for sex and decade based on a previous study (Table 1) [30]. During the 400-meter walk test, subjects are asked to walk as quickly as they could for 20 laps of a 20-meter course. This cut-off was selected to avoid a ceiling effect for the function outcome and to keep the study clinically relevant to mobility-limited older adults with symptomatic knee OA, the group that was unable to tolerate land-based power training [15, 31]. Subjects were recruited through advertisements posted in local businesses, information booths at local senior center events, and electronic mail sent to individuals affiliated with the University of Iowa. This protocol also was registered at clinicaltrials.gov as study NCT00904319.

Volunteers were asked “Do you have health problems that affect your walking or ability to exercise such as severe back pain, heart disease, diseases of the muscles or nerves, or problems with your eyesight that affect your walking?” and excluded if they reported causes for mobility limitation other than knee symptoms. No participants were unwilling to be in a 1.2-meter deep pool, or had a history of bilateral knee replacement, lower limb amputation, myocardial infarction or stroke in the past year, lower limb surgery in the last six months that affected walking ability, concurrent participation in another research study, or medical conditions that affected walking ability or ability to follow the protocol (e.g., Alzheimer's or other type of dementia, multiple sclerosis, Parkinson's disease, severe cardiovascular disease, congestive heart failure, severe dysrhythmias, severe emphysema, severe asthma, skin disease that would be adversely affected by aquatic exposure, inability to attend visits, or understand instructions). Those who met eligibility criteria other than mobility limitation were invited to a clinical visit, where volunteers participated in an institutional review board-approved informed consent process and then 400-meter walk time was assessed. Those who met inclusion criteria were scheduled for an initial pool visit and given a study description form for their usual physician to review and sign if the volunteer was medically safe to participate in the aquatic power training protocol.

### 2.2. Intervention

The aquatic power training program involved two training sessions per week of one hour duration for six weeks in a 1.2-meter deep therapy pool, heated to approximately 34.5° to 36° Celsius. A maximum of three subjects were trained at one time by one of two exercise specialists certified in the aquatic power training protocol. The subjects were encouraged to perform a total of eight exercises (Table 2), completing three sets of 10 repetitions each, performing each repetition as quickly as possible in the water. The specific exercises were selected to increase power in muscles affecting the knee joint, while observing principles of muscle overload, periodicity, progression, and specificity of training. Exercise form was taught to the subjects with a specialist in the water emphasizing ideal form with hands-on assistance if needed. At the beginning of each session, subjects were asked to rate their knee pain and asked whether any changes had been made in the their knee OA treatment since the previous appointment. Each session began with a 3–5 minute warmup that consisted of walking forwards and backwards in the pool, followed by step-ups on pool stairs or underwater risers (Speedo Aquatic Step, Speedo USA, Los Angeles, CA), step-downs on pool stairs or risers, standing row with foam aquatic dumbbells (intended to enable core muscle engagement necessary for power training during a lower limb rest period), posterolateral leg lifts, hip abduction and adduction leg lifts, calf raises (bilateral plantarflexion), and toe raises (bilateral dorsiflexion). The underwater pool staircase was used when a single subject was in the pool and underwater risers were used when there were multiple subjects training simultaneously. The subjects were told to perform the concentric phase of each repetition as rapidly as possible with a slow eccentric phase.

The exercise specialist provided verbal and tactile cues both to ensure proper form for each exercise and to encourage rapid contractions for power training. Between each exercise set, subjects walked 2.4 meters forwards and backwards as quickly as possible in chest-deep water. The exact level of submersion in water depended on the subject's height. At the conclusion of each training session, subjects were asked to complete 3–5 minutes of aquatic walking forwards and backwards as a cool-down before exiting the pool. Subjects' Borg ratings of perceived exertion (RPE) on a scale of 6 to 20 were monitored after each set of repetitions. If a participant had reported an RPE ≥17, then the subject would have been asked to rest until the RPE went back to 13. However, no subject reported an RPE greater than 14 during the study. Specialists recorded subject attendance, number of repetitions of each exercise performed, and presence of knee pain prior to and following each session. Participants' ratings of knee pain severity were assessed during training sessions for safety and to adjust the exercise intensity to avoid exacerbating symptoms.

Attempts to optimize compliance and retention included providing reminder phone calls as needed prior to appointments, compensation for attending study visits, and parking vouchers or bus passes at each study visit. 

### 2.3. Outcome Measurements

**Figure 1:**
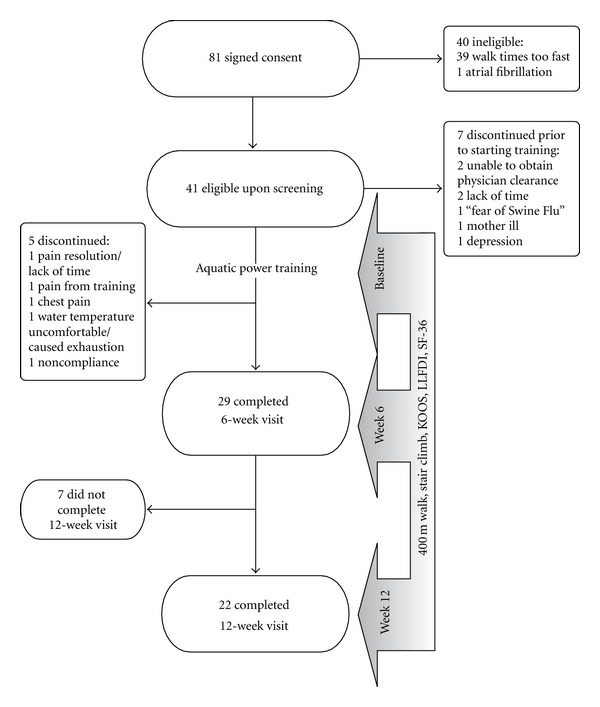
Enrollment and progression of subjects and timing of study measurements.

Tolerability (primary outcome measure) was assessed with ratings of knee pain and exertion, need for modification of the protocol, and attendance. Symptomatic and physical functional outcome measurements were conducted at baseline, following 6 weeks of training and again 6 weeks after completion of training to assess for durability of effects (12 weeks after baseline) as shown in Figure 1.

#### 2.3.1. Stair Climb Power

Subjects were instructed to safely ascend a standard 8-stair flight (total vertical distance = 1.441 meters), with handrails on both sides as quickly as possible. If necessary for balance, the handrails could be used on either side. Timing was started when the subject initiated foot movement to begin stair ascent and was stopped when both feet arrived on the top (eighth) step. Time was recorded to the nearest 0.01 second. Times for two trials, attempted on the same day, were averaged. The reliability for this test has been reported to be excellent (ICC = 0.97) [32]. Stair climb power (Watts) was calculated as the product of gravitational force (Newtons) and vertical distance (meters) divided by time (seconds) [33].

#### 2.3.2. Performance-Based Functional Limitation

The long distance corridor walk (LDCW) is a measure of timed gait during an unassisted walk and is sensitive to changes in community mobility. The protocol was based on the protocol used in the Health, Aging, and Body Composition (Health ABC) Study and the Osteoarthritis Initiative [34]. An advantage to the LDCW is that if a subject is unable to walk 400 meters, gait speed can still be estimated from a 2-minute walk.

#### 2.3.3. Self-Reported Functional Limitation

Self-reported difficulty with physical activity was measured using the Late Life Function and Disability Instrument: Function Component (LLFDI, Boston University, Boston, MA) [35]. The basic lower limb subscore was the primary measure of functional limitation.

#### 2.3.4. Knee Pain and Knee-Related Activities of Daily Living and Quality of Life

The knee injury and osteoarthritis outcome score (KOOS) is an extension of the Western Ontario and McMaster Universities Osteoarthritis Index (WOMAC), the most commonly used outcome instrument for assessment of patient-relevant treatment effects in OA. This instrument has been found to be a reliable, and responsive measure in older adults with knee OA, and sensitive to changes in pain and knee-related activities of daily living and quality of life [36]. This study utilized the knee pain, activities of daily living, and quality of life subscales.

#### 2.3.5. Bodily Pain

In addition to assessing for knee-specific pain that might relate to the exercise protocol, overall pain that might be modified by the aquatic environment was assessed with the SF-36, a well-validated tool that combines the dimensions of impairments and functional limitations [37]. Nine of the ten items on the SF-36 function subscale examine a dimension of lower limb function that can be affected by knee pain. The responsiveness is reportedly similar to the physical function subscales of the arthritis-specific arthritis impact measurement scale [38] and the Knee Society's Clinical Rating System for knee OA [39]. Bodily pain was the subscale of importance in measuring impairment for this study. 

### 2.4. Analytic Methods

Outcome measures were continuous. Tolerance of the intervention was assessed descriptively for pain and exertion ratings, modifications, and attendance. Tests of normality were performed, followed by construction of linear mixed models for repeated measures to test for changes in outcome measures across time points. Dunnett-Hsu's method was used to adjust for multiple comparisons in comparing each of the followup measurements (Week 6 and Week 12) with the baseline measurement. Symptomatic and functional outcome measures were summarized with least squares means and standard errors (SE) as well as absolute ranges. Effect sizes were calculated as the mean difference in the parameter between time points divided by the standard deviation of the mean difference in that parameter. Statistical analyses were conducted using SAS 9.2 with an alpha level of 0.05. 

### 2.5. Sample Size and Power

Although designed to assess tolerability, this study also was initiated to obtain evidence regarding whether aquatic power training would improve muscle power. In a prior study of land-based power training [13], the mean ± standard deviation (SD) for the change in lower limb power (at 50% 1 RM) was 25.2 ± 39.1 W. To detect this magnitude of difference in lower limb power would require a minimum of 27 subjects for 95% power (for a one-sided test with alpha = 0.05). Considering the potential for subject discontinuation, we planned to recruit 35 subjects to optimize the chances of maintaining this level of statistical power to detect a significant difference between the pre- and postintervention lower limb power.

## 3. Results

Figure 1 illustrates number of participants screened, recruited and enrolled. Prior to the initial training session, 7 subjects discontinued participation due to: inability to obtain medical clearance to participate (2), lack of time (2), family emergency (1), fear of swine flu (1), and depression (1). Of the 34 subjects who started the study, 5 subjects discontinued participation: pool temperature too warm (1), resolution of knee pain and lack of time (1), worsening of knee pain (1), chest pain (1), and failure to attend at least half of the visits (1). This left 29 subjects (18 women) who completed 6 weeks of aquatic training and for whom symptomatic and functional data were available. Mean age was 66.7 ± 9.0 years (range = 53–87 years). Seven subjects did not return for the 12-week follow-up assessment. Reasons for this were subject vacation plans (2), inability of study staff to reach the subject (1), subject did not return calls to schedule the assessment (1), subject illness (1), subject medical procedure (1), and repetitive scheduling conflicts (1). During the study period, no subjects reported seeking health care for knee OA. Four subjects made minor changes to their treatment: taking ginger (1), gin-soaked raisins (1), or changing NSAID dose (2). One subject also took Tylenol on one occasion during the study period. Otherwise, subjects reported no changes in the management of their pain.

### 3.1. Tolerance

Overall attendance at training visits was 92.2% (hours attended/hours scheduled), with 15 participants attending all 12 visits, the median number of visits attended (interquartile range 11-12 visits). 

Twenty-one of 29 subjects completed the study per protocol. The remaining 8 required modification of the protocol for tolerance. Two shorter subjects performed the exercises on the underwater staircase due to feeling uncomfortable with the 1.2-meter pool depth. Other subjects who required a modification to the protocol reported Achilles tendon pain (1), knee pain (2), or muscle pain (1) when attempting the standard protocol or could not complete the exercises at a velocity judged by the trainer to be consistent with power training. In such cases, trainers reduced the number of repetitions and sets. However, one subject required an increase in repetitions due to insufficient intensity. 

The ratings on the verbal analogue scale of knee pain prior to and following each aquatic training session were normally distributed. The mean ± SD pretraining and posttraining pain ratings were 3.1 ± 2.2 and 2.8 ± 2.2, respectively, with a reduction in pain of 0.3 ± 0.7 between initiation and completion of the sessions. 

### 3.2. Symptomatic and Functional Outcomes at 6 Weeks

Baseline measurement results and change at each followup time point are summarized in Table 3. There was an improvement in stair climb time (*P* = 0.0036) and stair climb power (*P* = 0.0075). In addition, activities of daily living and quality of life as reported on the KOOS improved by 6.9–7.4 points between baseline and completion of the 6-week intervention (all *P* < 0.05). There also was a statistically significant 10.5-point improvement on the bodily pain scale of the SF-36. However, neither 400-meter walk time nor report of functional status on the basic lower limb subscore of the LLFDI significantly changed over the period of the intervention. 

### 3.3. Durability of Effects on Impairments and Functional Limitations at 12 Weeks

Six weeks after completion of the aquatic intervention, participants continued to demonstrate improvement in stair climb time and power, as well as in knee pain, activities of daily living, quality of life, and bodily pain. However, the effects on bodily pain and quality of life were attenuated in the context of 7 of the 29 subjects failing to return for the 12-week visit. There continued to be no significant effect on 400-meter walk time and basic lower limb function score on the LLFDI in comparison with baseline measures.

## 4. Discussion

Overall, the results of this study demonstrated feasibility and tolerance of an aquatic-based power training intervention and potential for an immediate beneficial effect on lower limb power. The detection of improved knee function (stair climb time, KOOS knee-specific activities of daily living and quality of life (QOL) and SF-36 bodily pain score) supports the need for a controlled trial to assess the independent effects of the aquatic power training program. The durability of these findings when assessed 6 weeks following the conclusion of training suggests that short-term participation could potentially provide longer-term benefits as significant effects were maintained without continuation of training. However, the primary relevance of the findings of this study relates to subjects' tolerance of the aquatic power training program, as well as the suitability of the eligibility criteria, outcomes, and intervention.

Despite the need to modify the protocol for some participants, the compliance of 92% (319 visits attended out of 348 total for 29 subjects), for the 6-week intervention time period, suggests that the intervention was well tolerated. The report of increased pain in only 4 of 34 subjects and retention of 29 of 34 subjects who started the program was considerably better than the rate of 50% in a land-based power training program that had similar eligibility criteria [15]. Although this was considered to be excellent for this group with symptomatic knee OA, and mobility limitations, the incidence of pain exacerbation might be reduced further through providing nonpharmacological or pharmacological modalities for pain management or slowing the rate of exercise progression, particularly regarding the velocity of the exercises. With regard to the need to modify the stepping exercises for 2 of the shorter participants, use of an adjustable-depth pool to accommodate people of differing heights may enhance tolerance of the exercise program.

The eligibility criteria were selected to specifically target subjects with knee OA with clinically relevant needs—those with daily pain and with mobility limitations. A total of 39 people with symptomatic OA, who were screened, were ineligible to take part in the study due to a walking speed that was above average for age and sex (Table 1) [30]. If those participants had not benefited from the intervention, it could be explained by lack of clinical need for power training due to a ceiling effect on physical function. Of the 34 subjects who began training, only 3 of them discontinued for reasons that related to the aquatic exercise program. Therefore, we believe that the eligibility criteria met the needs of identifying an appropriate clinical window—that is, including subjects with potential for improvement, but who were able to tolerate the intervention. These eligibility criteria also enabled the study to be generalizable to patients with a clinical need for rehabilitation.

As the intervention was designed with the intent to improve mobility, the 400-meter walk test was selected as a suitable primary outcome measure for mobility. However, this test may not be the optimal outcome measure for this aquatic training program. In retrospect, these findings are consistent with the principle of specificity of training, in which exercises that closely approximate the goal functional activity are the most effective in improving physical performance during that activity. The exercises involved stepping motions as well as targeting muscles necessary for stepping, but did not focus on walking. Similarly, in a land-based study of adults over age 70, power training improved muscle power but not walk time compared with a walking program [11]. Likewise, the present power training intervention resulted in significant improvement in both stair climb time and muscular power but not 400-meter walk times (functional improvement). Additionally, our findings are consistent with those of another aquatic therapy interventional study, in which investigators reported knee pain but not walking speed improved in an aquatic compared with a land-based intervention group [25]. Therefore, if the intervention remained unaltered, in future research involving this aquatic power training program, it would be more appropriate to select a lower limb power or stepping test as the primary outcome measure. 

Alternatively, underwater treadmill training could be added to increase the specificity for targeting gait speed. As the intervention was originally intended to improve gait speed in older adults with symptomatic knee OA and mobility limitations, water walking was included between the exercises. However, this exposure was of an insufficient training intensity to elicit a detectable improvement in gait speed. Therefore, we speculate that the increased intensity involved with underwater treadmill training might be better suited to achieving the goal of improving gait speed while maintaining the partial weight-bearing necessary for tolerance of the intervention.

As impairments in lower limb muscle power have been associated with mobility limitations [9] and correlate better with functional limitations in older adults than does strength [7, 10–12], the intervention in the current study was designed to take advantage not only of hydrostatic, but also hydrodynamic principles. Rather than using a land-based program transferred to an aquatic environment and using increasing repetitions to advance the exercises [23], our program advanced the difficulty level through reducing buoyancy (decreasing depth of immersion reduces the upward force due to reducing the volume of water being displaced-thereby increasing weight bearing), increasing surface area exposed to the direction of movement (increasing drag), increasing speed of movement, and generating turbulent flow through moving closer to the pool wall. 

Although the intervention may not have been well targeted towards walking, it did demonstrate potential for eliciting improvements in symptoms and quality of life. In a prior 6-week study of adults with a similar mean age and meeting similar inclusion criteria (ACR criteria for OA and frequent pain), Hinman et al. found that an aquatic physical therapy intervention resulted in improved WOMAC (a subset of KOOS) [36] symptoms, function, and quality of life as well as greater distance walked over 6 minutes in individuals with hip or knee OA [17]. The respective effect sizes for improvement in KOOS pain (0.44 versus 0.28), quality of life (0.69 versus 0.17), and walk time (0.06 versus 0.01) were somewhat higher in the current study in comparison with that of Hinman et al. [17], and this may relate to the presence of a control group in the prior study. Similar to that study, our intervention was based on the hydrostatic and hydrodynamic principles of the aquatic environment with an emphasis on proper form during functional exercises. However, the trainers in our study were not physical therapists, nor did they have prior experience with aquatic exercises, demonstrating that even aquatic training led by less experienced exercise trainers may elicit benefits for community-dwelling adults with symptomatic knee OA and mobility limitations.

When assessing improvement in impairments and functional limitations, it is necessary to confirm that changes detected are not merely statistically significant, but also clinically meaningful and that the results are generalizable. Considering that the minimum clinically important difference (MCID) for improvement on the WOMAC pain scale (a subset of the KOOS) has been reported to be 2.1 points [40], the reduction in knee pain of 5.5 points on the KOOS in this study suggests clinically meaningful improvement. In addition, the concordant 11-point improvement on the SF-36 bodily pain scale also exceeded the 3.3 to 7.8 point MCID for improvement reported for the SF-36 bodily pain domain [41, 42]. In addition, the baseline characteristics of the participants—400-meter walk time slower than the median for the age group and significant daily knee pain—demonstrate that the subjects enrolled were representative of the target population of interest, older adults with symptomatic knee OA and mobility limitations. Therefore, it appears that the clinically important improvements may be generalizable to others with similar characteristics.

A second important finding of this study was that the significant improvements were maintained 6 weeks following discontinuation of the intervention, suggesting that the intervention may not need to be continuous to confer some residual benefits. This result is consistent with the previous report by Hinman et al., in which improvements in symptoms, function, and mobility were maintained 6 weeks after completion of the supervised intervention. The sustained effect in the study of Hinman et al. may have related to the fact that 84% of participants continued the intervention independently. Although participants were allowed to continue training during the 6 weeks following completion of the intervention in the present study, no subjects elected to continue the intervention—possibly due to the location of the pool in the medical center being inconvenient for subjects or due to the high water temperature. Interestingly, despite the lack of continued participation, subjects demonstrated sustained reduction in impairments and functional limitations.

The principal limitation of this pilot study was the absence of a control group. This study was conducted to assess the feasibility and tolerance of power training in an aquatic environment among adults with symptomatic knee OA, rather than primarily to assess efficacy. Because there was no control group, in addition to the potential for the power training intervention to have accounted for the results, an alternative possibility is that nonspecific effects of the aquatic environment may have contributed to the results. Given the universally soothing effect of warm water, it is possible that the reduction in pain may be partially attributable to (a) the effects of buoyancy on decreasing joint loading, (b) specific heat enabling increased joint range of motion, and/or (c) vasodilatation enhancing nutrition of joint tissues and clearance of edema and metabolic waste products [43, 44]. Another alternative explanation for the improvements in the outcomes detected could relate to attention provided by the trainer (i.e., placebo effect).

Another limitation was the lack of a quantitative assessment of the degree to which subjects were power training. Interpretation of the results would benefit from incorporating an objective measure of the force and velocity of motion during the aquatic exercises. Our observation suggested that 21 of the 29 subjects engaged in exercise of sufficient velocity to be considered power training. Those who did not engage in what we believed to be true power training did not do so due to concern for exacerbation of joint pain. If the intervention were individualized, a progressive exercise program, in which, subjects who have difficulty with the power training start at a low intensity (below the threshold for pain provocation) and progressively increase the intensity to a level appropriate for power training, could potentially enable tolerance in a greater proportion of subjects. Lastly, although we found that 6 weeks following completion of the protocol, many of the benefits were sustained, no subjects elected to continue following completion of the supervised training. In order to assess whether continuation may result in greater benefits, future studies should incorporate a motivational intervention. 

## 5. Conclusion

In conclusion, our data suggest that a 6-week aquatic rehabilitation program is both feasible and well tolerated by adults over age 50 with daily knee pain, clinical knee OA, and mobility limitations. Furthermore, this program may result in improved lower limb muscle power, symptoms, activities of daily living, and quality of life, but may not improve walking speed or self-reported lower limb function. The results suggest that benefits persist for at least 6 weeks following discontinuation of the program. Future studies should include a comparison group, an objective measure of the power training intensity, a greater duration of follow-up, and eventually a transition from aquatic to land-based training.

## Figures and Tables

**Table 1:** Mobility limitation inclusion criteria [30].

Sex	Decade	400 m walk time (sec)
Men	50's	>250.2
	60's	>289.9
	70's+	>290.8

Women	50's	>315.9
	60's	>305.2
	70's+	>292.5

**Table 2:** Aquatic power training exercises.

Exercise	Sets	Repetitions
Step-ups on the pool stairs or underwater riser	3	10 on each leg
*Walking forward and backwards*	2	
Step-downs on pool stairs or underwater risers	3	10 on each leg
*Walking forward and backwards*	2	
Row with foam water dumbbells	3	10
*Walking forward and backwards*	2	
Hip extension leg lifts	3	10 on each leg
*Walking forward and backwards*	2	
Hip abduction	3	10
*Walking forward and backwards*	2	
Hip adduction	3	10
*Walking forward and backwards*	2	
Plantar flexion heel raises	3	10
Calf stretches—30 seconds each stretch	2	
*Walking forward and backwards*	2	
Dorsiflexion toe raises	3	10

**Table 3:** Baseline and weeks 6 and 12 changes (LS mean ± SE).

Variable	Week 0 (range)	Week 6 change	Effect size	Adjusted *P* value*	Week 12 change	Adjusted *P* value*
400-meter walk time (sec)	355.6 ± 10.5 (257.8–468.8)	1.2 ± 7.1	0.06	0.9805	−3.3 ± 7.8	0.8828
Stair climb time (sec)	5.7 ± 0.3 (3.4–9.7)	−0.5 ± 0.2	−0.56	0.0036	−0.6 ± 0.2	0.0013
Stair climb power (W)	221.6 ± 18.6	22.3 ± 7.1	0.62	0.0075	27.6 ± 8.5	0.0051
LLFDI: basic lower limb function	64.5 ± 1.7 (47.8–88.0)	1.8 ± 1.2	0.32	0.2279	1.6 ± 1.3	0.3657
KOOS: knee pain subscale	60.8 ± 3.1 (53.0–87.0)	5.3 ± 2.5	0.44	0.0743	6.4 ± 2.8	0.0496
KOOS: knee-related activities of daily living	66.5 ± 2.6 (41.8–91.2)	6.9 ± 2.4	0.56	0.0107	8.1 ± 2.6	0.0070
KOOS: knee-related quality of life	39.4 ± 3.4 (12.5–68.8)	7.4 ± 2.4	0.69	0.0068	7.0 ± 2.7	0.0224
SF36: bodily pain score	57.1 ± 3.5 (10.0–90.0)	10.5 ± 3.2	0.69	0.0040	8.3 ± 3.6	0.0453

^∗^Dunnett-Hsu adjustment; for these repeated measures analyses, 81 observations were used, with each participant contributing data at a maximum of 3 time points.

## References

[1] March LM, Bachmeier CJM (1997). Economics of osteoarthritis: a global perspective. *Bailliere’s Clinical Rheumatology*.

[2] Hootman JM, Helmick CG (2006). Projections of US prevalence of arthritis and associated activity limitations. *Arthritis and Rheumatism*.

[3] Davis MA (1988). Epidemiology of osteoarthritis. *Clinics in Geriatric Medicine*.

[4] Guccione AA (1994). Arthritis and the process of disablement. *Physical Therapy*.

[5] Izquierdo M, Ibañez J, Gorostiaga E (1999). Maximal strength and power characteristics in isometric and dynamic actions of the upper and lower extremities in middle-aged and older men. *Acta Physiologica Scandinavica*.

[6] Lauretani F, Russo CR, Bandinelli S (2003). Age-associated changes in skeletal muscles and their effect on mobility: an operational diagnosis of sarcopenia. *Journal of Applied Physiology*.

[7] Skelton DA, Greig CA, Davies JM, Young A (1994). Strength, power and related functional ability of healthy people aged 65–89 years. *Age and Ageing*.

[8] Suzuki T, Bean JF, Fielding RA (2001). Muscle power of the ankle flexors predicts functional performance in community-dwelling older women. *Journal of the American Geriatrics Society*.

[9] Bean JF, Kiely DK, Herman S (2002). The relationship between leg power and physical performance in mobility-limited older people. *Journal of the American Geriatrics Society*.

[10] Earles DR, Judge JO, Gunnarsson OT (1997). Power as a predictor of functional ability in community dwelling older persons. *Medicine and Science in Sports and Exercise*.

[11] Earles DR, Judge JO, Gunnarsson OT (2001). Velocity training induces power-specific adaptations in highly functioning older adults. *Archives of Physical Medicine and Rehabilitation*.

[12] Foldvari M, Clark M, Laviolette LC (2000). Association of muscle power with functional status in community-dwelling elderly women. *Journals of Gerontology—Series A*.

[13] Bean JF, Herman S, Kiely DK (2004). Increased velocity exercise specific to task (invest) training: a pilot study exploring effects on leg power, balance, and mobility in community-dwelling older women. *Journal of the American Geriatrics Society*.

[14] Guralnik JM, Ferrucci L, Simonsick EM, Salive ME, Wallace RB (1995). Lower-extremity function in persons over the age of 70 years as a predictor of subsequent disability. *The New England Journal of Medicine*.

[15] Segal N, Clearfield J, Yack H, Torner J, Wallace R Improving mobility for older adults with symptomatic knee osteoarthritis: effects of gait and power training.

[16] Silva LE, Valim V, Pessanha APC (2008). Hydrotherapy versus conventional land-based exercise for the management of patients with osteoarthritis of the knee: a randomized clinical trial. *Physical Therapy*.

[17] Hinman RS, Heywood SE, Day AR (2007). Aquatic physical therapy for hip and knee osteoarthritis: results of a single-blind randomized controlled trial. *Physical Therapy*.

[18] Lin SYC, Davey RC, Cochrane T (2004). Community rehabilitation for older adults with osteoarthristis of the limb: a controllefd clinical trial. *Clinical Rehabilitation*.

[19] Suomi R, Lindauer S (1997). Effectiveness of arthritis foundation aquatic program on strength and range of motion in women with arthritis. *Journal of Aging and Physical Activity*.

[20] Pöyhönen T, Sipilä S, Keskinen KL, Hautala A, Savolainen J, Mälkiä E (2002). Effects of aquatic resistance training on neuromuscular performance in healthy women. *Medicine and Science in Sports and Exercise*.

[21] Valtonen A, Pöyhönen T, Sipilä S, Heinonen A (2010). Effects of aquatic resistance training on mobility limitation and lower-limb impairments after knee replacement. *Archives of Physical Medicine and Rehabilitation*.

[22] Valtonen A, Poyhonen T, Sipila S, Heinonen A (2011). Maintenance of aquatic training-induced benefits on mobility and lower-extremity muscles among persons with unilateral knee replacement. *Archives of Physical Medicine and Rehabilitation*.

[23] Foley A, Halbert J, Hewitt T, Crotty M (2003). Does hydrotherapy improve strength and physical function in patients with osteoarthritis—a randomised controlled trial comparing a gym based and a hydrotherapy based strengthening programme. *Annals of the Rheumatic Diseases*.

[24] Lund H, Weile U, Christensen R (2008). A randomized controlled trial of aquatic and land-based exercise in patients with knee osteoarthritis. *Journal of Rehabilitation Medicine*.

[25] Wyatt FB, Milam S, Manske RC, Deere R (2001). The effects of aquatic and traditional exercise programs on persons with knee osteoarthritis. *The Journal of Strength & Conditioning Research*.

[26] Bartels EM, Lund H, Hagen KB, Dagfinrud H, Christensen R, Danneskiold-Samsøe B (2007). Aquatic exercise for the treatment of knee and hip osteoarthritis. *Cochrane Database of Systematic Reviews*.

[27] Fransen M, Nairn L, Winstanley J, Lam P, Edmonds J (2007). Physical activity for osteoarthritis management: a randomized controlled clinical trial evaluating hydrotherapy or Tai Chi classes. *Arthritis Care and Research*.

[28] Arnold CM, Faulkner RA (2010). The effect of aquatic exercise and education on lowering fall risk in older adults with hip osteoarthritis. *Journal of Aging and Physical Activity*.

[29] Altman R, Asch E, Bloch D (1986). Development of criteria for the classification and reporting of osteoarthritis. Classification of osteoarthritis of the knee. *Arthritis and Rheumatism*.

[30] Segal NA, Yack HJ, Brubaker M, Torner JC, Wallace R (2009). Association of dynamic joint power with functional limitations in older adults with symptomatic knee osteoarthritis. *Archives of Physical Medicine and Rehabilitation*.

[31] Marsh AP, Miller ME, Saikin AM (2006). Lower extremity strength and power are associated with 400-meter walk time in older adults: the InCHIANTI study. *Journals of Gerontology—Series A*.

[32] Cuoco A, Callahan DM, Sayers S, Frontera WR, Bean J, Fielding RA (2004). Impact of muscle power and force on gait speed in disabled older men and women. *Journals of Gerontology—Series A*.

[33] Bean JF, Kiely DK, LaRose S, Alian J, Frontera WR (2007). Is stair climb power a clinically relevant measure of leg power impairments in at-risk older adults?. *Archives of Physical Medicine and Rehabilitation*.

[34] Simonsick EM, Montgomery PS, Newman AB, Bauer DC, Harris T (2001). Measuring fitness in healthy older adults: the health ABC long distance corridor walk. *Journal of the American Geriatrics Society*.

[35] Sayers SP, Jette AM, Haley SM, Heeren TC, Guralnik JM, Fielding RA (2004). Validation of the late-life function and disability instrument. *Journal of the American Geriatrics Society*.

[36] Roos EM, Toksvig-Larsen S (2003). Knee injury and osteoarthritis outcome score (KOOS)—validation and comparison to the WOMAC in total knee replacement. *Health and Quality of Life Outcomes*.

[37] Ware JE (2000). SF-36 health survey update. *Spine*.

[38] Nevitt MC (2002). Obesity outcomes in disease management: clinical outcomes for osteoarthritis. *Obesity Research*.

[39] Kantz ME, Harris WJ, Levitsky K, Ware JE, Davies AR (1992). Methods for assessing condition-specific and generic functional status outcomes after total knee replacement. *Medical Care*.

[40] Zhao SZ, McMillen JI, Markenson JA (1999). Evaluation of the functional status aspects of health-related quality of life of patients with osteoarthritis treated with celecoxib. *Pharmacotherapy*.

[41] Angst F, Aeschlimann A, Stucki G (2001). Smallest detectable and minimal clinically important differences of rehabilitation intervention with their implications for required sample sizes using WOMAC and SF-36 quality of life measurement instruments in patients with osteoarthritis of the lower extremities. *Arthritis Care and Research*.

[42] Strand V, Kelman A (2004). Outcome measures in osteoarthritis: randomized controlled trials. *Current Rheumatology Reports*.

[43] Prins J, Cutner D (1999). Aquatic therapy in the rehabilitation of athletic injuries. *Clinics in Sports Medicine*.

[44] Wilder R, Cole A, Becker B (1998). *Aquatic Strategies for Athletic Rehabilitation*.

